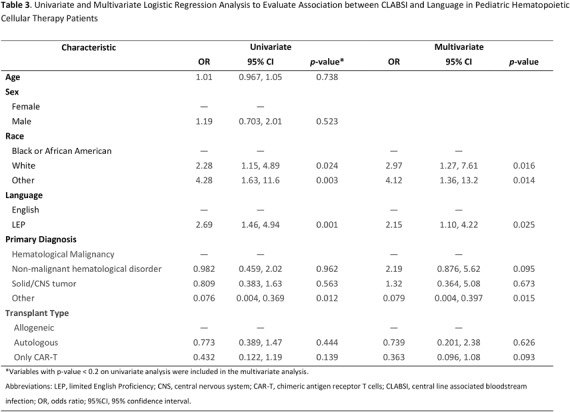# Does Language Barrier Impact CLABSI Risk in Pediatric Hematopoietic Cellular Therapy Patients?

**DOI:** 10.1017/ash.2025.340

**Published:** 2025-09-24

**Authors:** Afreen Abraham, Kelly Jennemann, Jose Amadeo Ferrolino, Ronald Dallas, Craig Gilliam, Gabriela Maron, Hana Hakim

**Affiliations:** 1 St. Jude Children’s Hospital and University of Tennessee; 2 St. Jude Children’s Research Hospital; 3 Department of Infectious Diseases, St. Jude Children’s Research Hospital

## Abstract

**Background:** Pediatric patients with underlying malignancies and hematological disorders, especially those undergoing hematopoietic cellular therapy (HCT) are at increased risk of developing central line-associated bloodstream infections (CLABSI). Despite the long-standing efforts to reduce CLABSI rates, healthcare disparities, particularly in minority populations, continue to contribute to preventable harm. The role of language barriers in CLABSI risk has not been well-explored, especially in pediatric HCT populations. This study aimed to evaluate the association between limited English proficiency (LEP) and CLABSI risk in pediatric HCT patients. **Methods:** This retrospective cohort study analyzed patients admitted to the transplant and cellular therapy unit of a pediatric oncology center between January 2021 and June 2024. Data collected from the electronic health records included patient demographics, preferred language, underlying primary diagnosis, types and dates of cellular therapy, and central line days (CLD) in the transplant unit. CLABSI events were identified through the hospital infection prevention and control surveillance database and classified as mucosal barrier injury laboratory confirmed bloodstream infection (MBI-LCBI) or non-MBI CLABSI based on NHSN definitions. CLABSI rates were calculated as events per 1,000 inpatient CLD and were compared between patients with LEP (defined as preferred language other than English) and those proficient in English. Multivariate regression model was used to evaluate the independent risk factors for CLABSI. **Results:** During the study period, 280 patients contributed a total of 12,325 CLD and 93 CLABSI events; of these 57 patients with LEP contributed 3,113 CLD and 29 CLABSI. The crude CLABSI rate in patients with LEP was significantly higher (9.32/1000 CLD) than that in English proficient patients (6.95/1000 CLD) **Conclusions:** This study highlights the increased risk of CLABSI in pediatric HCT patients with language barriers, the risk of which likely extends beyond this patient group. Addressing these barriers through novel equitable strategies, such as language support services and culturally competent care, is crucial for reducing healthcare disparities, improving clinical outcomes and promoting a more inclusive healthcare environment for diverse patient groups.

**Figure:**
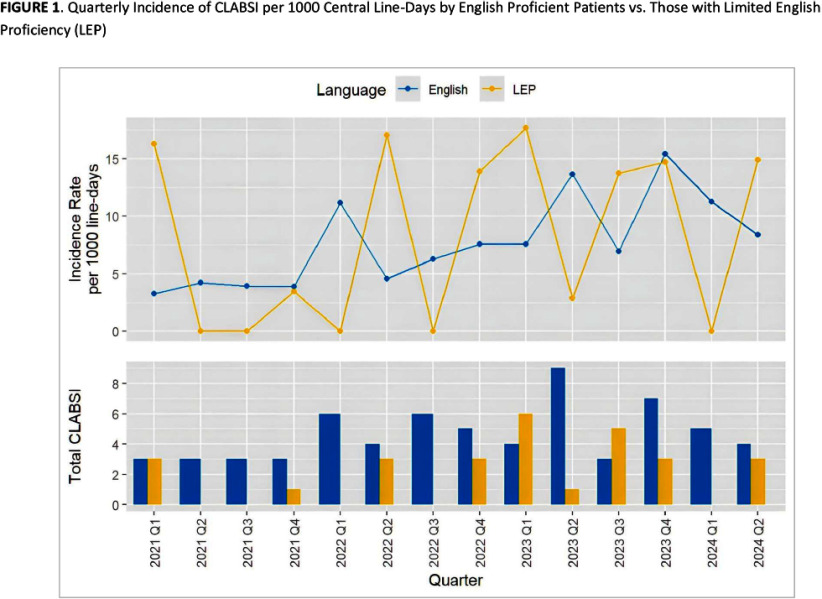

**Figure:**
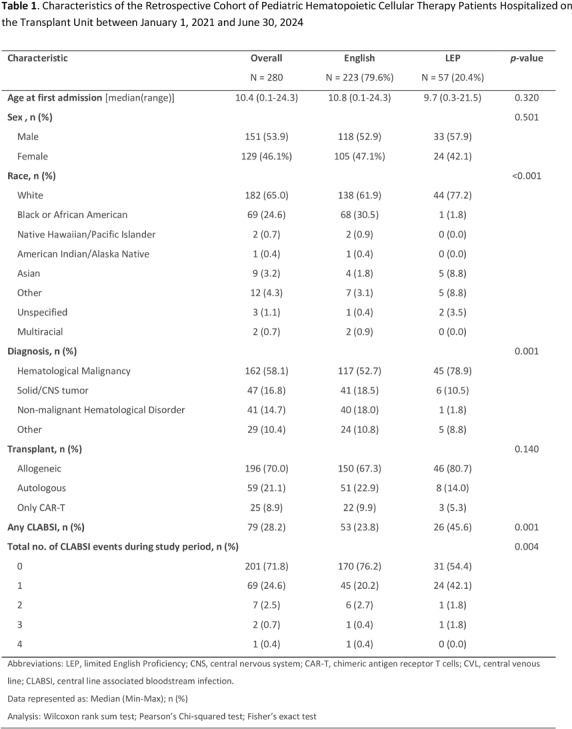

**Figure:**
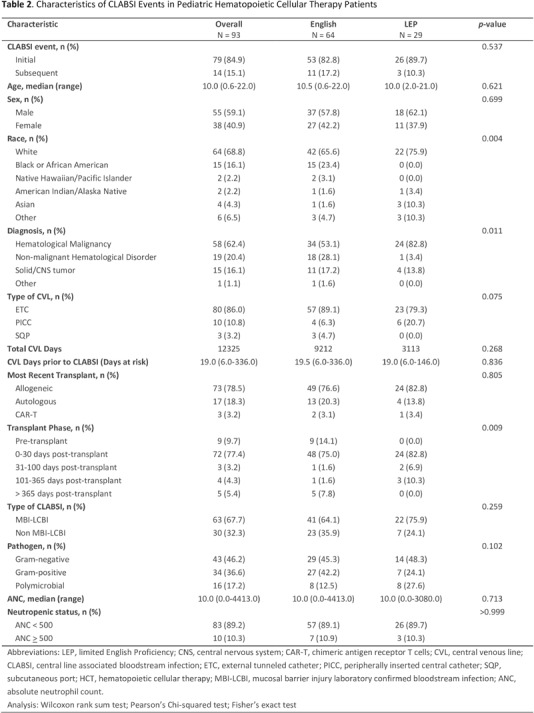

**Figure:**